# CRISPR-Cas influences the acquisition of antibiotic resistance in *Klebsiella pneumoniae*

**DOI:** 10.1371/journal.pone.0225131

**Published:** 2019-11-20

**Authors:** Natalie A. Mackow, Juntao Shen, Mutayyaba Adnan, Aisha S. Khan, Bettina C. Fries, Elizabeth Diago-Navarro

**Affiliations:** 1 Department of Medicine, Infectious Disease Division, Stony Brook University, Stony Brook, New York, United States of America; 2 School of Life Science and Biotechnology, Dalian University of Technology, Dalian, PR China; 3 Department of Molecular Genetics and Microbiology, Stony Brook University, Stony Brook, New York, United States of America; Academia Sinica, TAIWAN

## Abstract

In the US Carbapenem resistance in *Klebsiella pneumoniae* (*Kp*) is primarily attributed to the presence of the genes *bla*_KPC-2_ and *bla*_KPC-3_, which are transmitted via plasmids. Carbapenem-resistant *Kp* (CR-*Kp*) infections are associated with hospital outbreaks. They are difficult to treat, and associated with high mortality rates prompting studies of how resistance is obtained. In this study, we determined the presence of CRISPR-Cas in 304 clinical *Kp* strains. The CRISPR-Cas system has been found to prevent the spread of plasmids and bacteriophages, and therefore limits the horizontal gene transfer mediated by these mobile genetic elements. Here, we hypothesized that only those *Kp* strains that lack CRISPR-Cas can acquire CR plasmids, while those strains that have CRISPR-Cas are protected from gaining these plasmids and thus maintain sensitivity to antimicrobials. Our results show that CRISPR-Cas is absent in most clinical *Kp* strains including the clinically important ST258 clone. ST258 strains that continue to be sensitive to carbapenems also lack CRISPR-Cas. Interestingly, CRISPR-Cas positive strains, all non-ST258, exhibit lower resistance rates to antimicrobials than CRISPR-Cas negative strains. Importantly, we demonstrate that the presence of CRISPR-Cas appears to inhibit the acquisition of *bla*_KPC_ plasmids in 7 *Kp* strains. Furthermore, we show that strains that are unable to acquire *bla*_KPC_ plasmids contain CRISPR spacer sequences highly identical to those found in previously published multidrug-resistance-containing plasmids. Lastly, to our knowledge this is the first paper demonstrating that resistance to *bla*_KPC_ plasmid invasion in a CRISPR-containing *Kp* strain can be reversed by deleting the CRISPR-cas cassette.

## Introduction

*Klebsiella pneumoniae* (*Kp*) is a gram-negative bacterium that predominately causes nosocomial infections including sepsis, urinary tract infections, and pneumonia. With increasing spread they now also cause community-acquired infectons. Carbapenem-resistant *Klebsiella pneumoniae* (CR-*Kp*) has increased in prevalence in the United States from 1.6% in 2001 to 10.4% in 2011 [1]. Treating CR-*Kp* is challenging. The combination of increased vulnerability of hospitalized patients with serious comorbidities, and inadequate treatment options explain overall 30-day mortality rates that are as high as 39–50% in bacteremic CR-*Kp* infected patients [2–7]. In the United States, resistance to carbapenem antibiotics in *Kp* strains is predominantly conferred by the two antibiotic resistance genes, *bla*_KPC-2_ and *bla*_KPC-3_, which encode class A carbapenem-hydrolyzing β-lactamases, the Klebsiella pneumoniae carbapenemases (KPC) are transfered through plasmids. In these KPC ß-lactamases convey resistance also against clavulanic acid, monobactams, and cephalosporins [8, 9]. The majority of clinical isolates of CR-*Kp* in the US belong to the multi-locus sequence type (MLST)-defined clone ST258 [4]. This clone emerged in the US in the 2000s and is now prevalent worldwide [3, 10]. Finally, numerous hospital outbreaks have been associated with the ST258 clone and its related variants making its investigation clinically imperative [11, 12].

While the presence of either *bla*_KPC-2_ or *bla*_KPC-3_ is likely not the only factor contributing to the success of ST258, their acquisition provides the bacteria with a significant advantage against antibiotic therapeutics. It is noteworthy that not all ST258 strains exhibit resistance to carbapenem. As transmission of *bla*_KPC_ genes is facilitated by plasmids, we analyzed the presence and function of CRISPR-Cas in carbapenem-sensitive (CS)-*Kp* and CR-*Kp* hospital strains. The CRISPR-Cas system is composed of *cas* genes followed by groupings of brief direct repeats and variable spacer regions that match infective agents of bacteria and archea, such as bacteriophages and plasmids [13–16]. This system has been found to prevent the spread of plasmids and bacteriophages [17–19], and therefore limit the horizontal gene transfer by these mobile genetic elements. For example, it was recently determined that hospital acquired, multidrug resistant strains of *Enterococcus faecium* lack CRISPR while community-acquired, drug sensitive strains of *E*. *faecium* contained CRISPR [20]. Additional studies demonstrated a significant association between antibiotic resistance in *E*. *faecium* strains and the absence of a CRISPR-Cas system, and correlation between decreased CRISPR-Cas and reduced defense against plasmid acquisition [21, 22]. CRISPR-Cas has therefore been compared in its function to an acquired immune system in bacteria.

We explored presence of the CRISPR-Cas systems in clinical *Kp* strains including those of the ST258 clonal background. Previous analysis on *Kp* genomes have identified presence of CRISPR-Cas, though its role in this species is yet to be defined [23, 24]. Until this study, little was known about the prevalence of CS-*Kp* strains and the predisposition of *Klebsiella* strains to become resistant to carbapenem antibiotics. While investigation of the overall prevalence of CRISPR-Cas in *Kp* has begun, the presence of CRISPR-Cas in CR versus CS-*Kp* and in the clinically important ST258 clone has not yet been studied. This project aimed to further elucidate the function of CRISPR-Cas in *Kp* immunity and examine the ST258 clonal background that has spread globally and has been highly associated with recent hospital outbreaks.

## Materials and methods

### Clinical isolates

Specimen collection was done under the approved IRB#648612 from Stony Brook University. No informed consent was obtained as samples were laboratory leftovers and deidentified. 304 hospital *Kp* isolates derived from bodily fluids from 265 patients were randomly collected from the microbiology laboratory at Stony Brook University Hospital (SBUH) between 2015–2016 (over a 14-month period) (S1 Table). The SBUH Microbiology laboratory regardless of the site of isolation kept isolates and our laboratory collect them. 83.3% were isolated from urine samples; 9.8% from respiratory samples, 4.6% from wounds and 2.2% from blood. This unbiased approach was chosen to permit identification of carbapenem sensitive ST258 strains. Sensitivity to all antibiotics was determined by the SBUH microbiology laboratory as part of standard diagnostic work up and obtained by us by review of medical records.

### Sequence typing of ST258 clone

Screening of ST258 clonal background was performed by PCR and sequencing of the *gap*A and *ton*B genes (S1 Fig). PCR was performed on colony DNA, analyzed by gel electrophoresis, and PCR products were purified using the Qiagen PCR purification kit. Purified PCR products were sent for Sanger sequencing to Genewiz^™^. If *gap*A and *ton*B results were *gap*A3 and *ton*B79, ST258 clonal background was confirmed by regular MLST typing of the rest of the gene array [25].

### CRISPR-Cas detection

Primers designed to detect *cas*1, *cse*1 and *cas*3 genes by PCR are listed in Table 1. Primers were designed using sequences provided by the webtool CRISPRFinder, which contained 111 CRISPR positive *K*. *pneumoniae* genomes and were representative of different CRISPR-Cas *cas* genes consistently seen in *Kp* CRISPR-Cas systems [16]. PCR was performed on colony DNA and results were analyzed by gel electrophoresis.

**Table 1:** Primers used in this study.

Gene/CRISPR-Cas system type	Primer	Sequence	Purpose
*cas*1	**FOR Cas1 K2**	GCTGTTTGTCAAAGTTACCCGCGAACTC	For Cas1 gene
*cas*1	**REV Cas1 K2**	GGTTTTGATCGCCTCATGAGTCACAGTTG	For Cas1 gene
*cse*1	**FOR Cse1 K2**	CAGTTTAACCGATATTTTCAGCCAGCCGG	For Cse1 gene
*cse*1	**REV Cse1 K2**	CATCAGTTAATTGCTGCTGTTGCTGACTTTCG	For Cse1 gene
*cas*3	**FOR Cas3 K2**	GGGTTTCGCTACAAAATCAACATGCCATCG	For Cas3 gene
*cas*3	**REV Cas3 K2**	CACGAGTTTTTTACGCTCATCAAACCAGAGC	For Cas3 gene
I-E CRISPR1	F-trp 1	CAGTTCCTGCAACCTGGCCT	For intact CRISPR1-Cas systems
I-E CRISPR1	R-Cas2 2	CTGGCAGCAGGTGATACAGC	
I-E CRISPR1	F-iap 3	CTGGCATAACGCCACCGG	For incomplete CRISPR1-Cas systems
I-E CRISPR1	R-cysH 4	GAGACCCGGTTCTTCGGGC	
I-E* CRISPR2	FU-Cas3 5	GTAGCGAAACCCTGATCAAGCG	For intact CRISPR-Cas systems IE*-CRISPR2
I-E* CRISPR2	R-L2 6	GCGCTACGTTCTGGGGATG	
I-E* CRISPR2	R-L2 6	GCGCTACGTTCTGGGGATG	For incomplete CRISPR-Cas systems IE*-CRISPR2
I E* CRISPR2	F-HP2 7	CGTCGCAAAACTCGACCAGA	
I-E* CRISPR3	F-HP1 8	GACGCTGGTGCGATTCTTGAG	For intact CRISPR-Cas systems IE*-CRISPR3
I-E* CRISPR3	RU-Cas2 9	CGCAGTATTCCTCAACCGCCT	
For CRISPR deletion	Down-70	GCCGCGATGGCATGTTGATTTTGTAGCGAAACCCTGATCAAGCGCCTCATCATATGAATATCCTCCTTAG	For I-E* CRISPR2 deletion
For CRISPR deletion	Up-70	CGCGCTACGTTCTGGGGATGACAAAAGCGTTTTACCCCCGGCTGCGGGCCGTGCAGGCTGGAGCTGCTTC	For I-E* CRISPR2 deletion

### Transformation assays

*bla*_KPC-2_ and *bla*_KPC-3_ plasmids were extracted by classic alkaline lysis extraction from previously reported CR strains M#34 and M#36 [4]. pPROBEKT plasmid (Km^R^, pVS1/p15a vector, with GFP-reporter gene) was used as a control for transformation [26]. 40 CS-Kp strains, 20 with and 20 without CRISPR-Cas, were randomly chosen for these studies and belong to different ST types as indicated by the combination of *gap*A and *ton*B alleles. CS-*Kp* strains were rendered electrocompetent and 100 ng of each of the plasmids was used in each of the transformation assays.

### Spacer analysis

**Fig 1:**
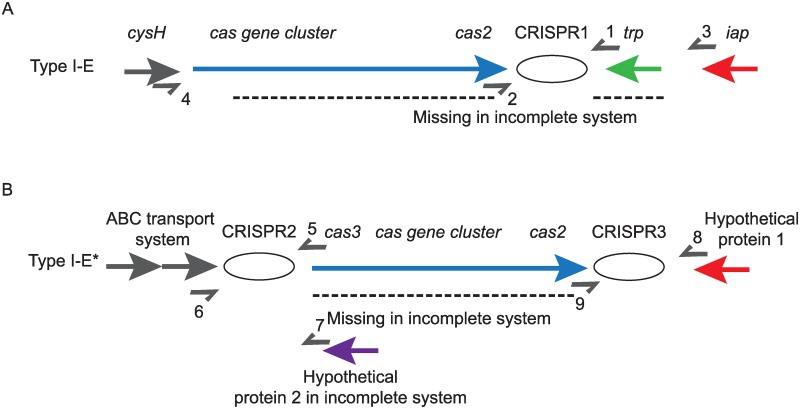
Primer locations with respect to CRISPR-Cas architecture. Names and localization of genes are depicted. Dotted lines represent DNA regions that may be deleted in certain strains. Primers are shown as half-arrows and numbers correspond to specific primers detailed in Table 1. CRISPR array regions are depicted with an ellipse. A) Type I-E CRISPR-Cas system architecture. A complete type I-E, CRISPR1 array will be amplified with primers 1 and 2; an incomplete type I-E CRISPR1 system will be amplified with primers 3 and 4. B) Type I-E* CRISPR-Cas system architecture. A complete type I-E*, CRISPR2 will be amplified with primers 5 and 6 and an incomplete type I-E*, CRISPR2 system will be amplified with primers 6 and 7. A complete type I-E* CRISPR3 system will be amplified with primers 8 and 9.

Based on previously published CRISPR-Cas architectures, primers were designed to amplify the CRISPR region in the two different CRISPR-Cas systems, type I-E and type I-E*, differentiated by flanking 3’ and 5’ genes (Fig 1) [22]. Primers used are listed in Table 1.

Genewiz^™^ was used to sequence PCR products. PCR sequences were analyzed with the CRISPRfinder tool to identify direct repeats and spacer sequences. Spacer sequences were then BLASTed using the NCBI non-redundant nucleotide database than includes all deposited genomes, plasmids or phages. BLAST search identified sequence homologies to previously reported DNA sequences.

### CRISPR deletion isogenic strains

To produce isogenic strains lacking CRISPR spacer regions, a Red/ET recombination system was used following manufacturer’s protocol (Quick and Easy *Escherichia coli* gene deletion kit; Gene Bridges, Heidelberg, Germany), but with modifications. Briefly, a linear DNA fragment with a chloranphenicol resistance and 70bp arms homologous to DNA upstream and downstream of I-E* CRISPR2 was amplified by PCR from the plasmid pCLF3 [27] with the primers listed in Table 1. The linear fragment was used to replace I-E* CRISPR2 regions in the 7 CRISPR-positive strains analyzed using the pRed/ET Tetracycline resistant plasmid. Positive transformants were confirmed by PCR using I-E* CRISPR2 specific primers.

## Results

### ST258 is significantly more carbapenem-resistant than other *Kp* clonal backgrounds

**Fig 2:**
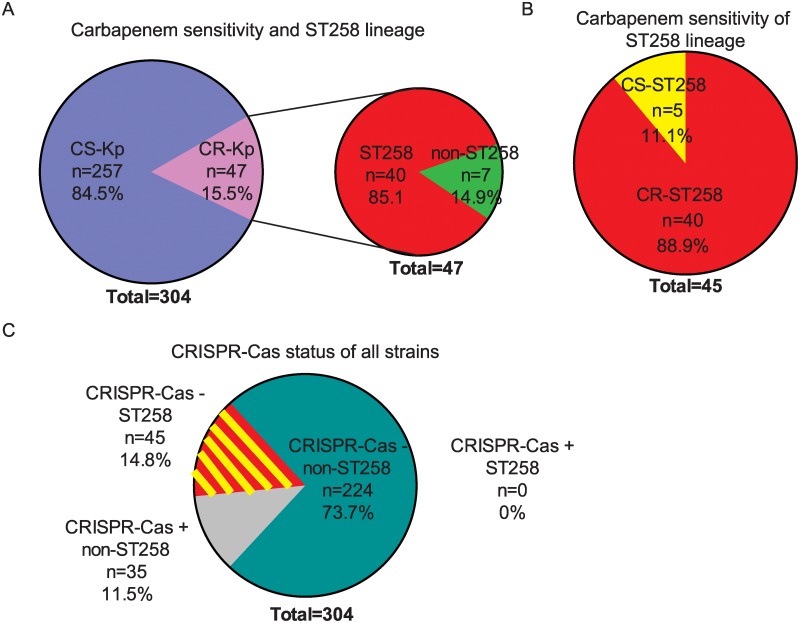
Characterization of clinical isolates. A) Of all *Kp* strains (n = 304), 15.5% were carbapenem-resistant (CR) (pink). Of those CR-*Kp*, 85.1% were ST258 strains (red), and 14.9% of CR strains were non-ST258 strains (green). B) Of 45 ST258 strains, 88.9% were CR (red) and 11.1% were carbapenem-sensitive (CS) (yellow). C) Analysis of the CRISPR-Cas content of all *Kp* strains (n = 304) identified only 11.5% CRISPR-Cas positive strains (grey), none of which were of the ST258 lineage. 88.5% of all *Kp* strains did not have CRISPR-Cas (green + red/yellow stripes), including all ST258 strains (both CR and CS, red/yellow stripes).

A PCR-based approach was designed that would allow identification of ST258 strains including those with maintained sensitivity to carbapenem antibiotics. We randomly collected 304 *Kp* strains (resistance unknown at collection time) that were identified by the microbiology laboratory of Stony Brook University Hospital (SBUH). PCR sequence typing for 2 genes (*gap*A, *ton*B) was performed to identify strains that strains belong to the ST258 clonal background. Those strains bearing *gap*A3 and *ton*B79 alleles were further typed by standard MLST (see Materials and methods section and S1 Fig). This approach identified a total of 45 ST258 strains (14.8%), 40 CR (89.2%) and 5 CS (11.1%) (Fig 2A). The ST258 clonal background accounted for 85.1% of all CR samples identified (Fig 2B). 257 Kp strains were identified as CS (84.5%), 5 of which were ST258. As expected *Kp* strains of the ST258 clonal background were significantly more likely to be CR compared with *Kp* strains of the non-ST258 clonal backgrounds (*p* < 0.0001, two-sided T test).

### All *Kp* strains with CRISPR-Cas were sensitive to carbapenems and had higher pan-sensitivity to other antibiotics

Next, *cas* genes were amplified by PCR to identify strains containing CRISPR-Cas systems (Materials and methods, Table 1). CRISPR-Cas was significantly less likely to be in strains of the ST258 clonal background (0/45) compared with strains of all other clonal backgrounds (35/259) (*p* = 0.0088, two-sided T test). MLST typing showed that CRISPR-Cas positive strains belonged to different clonal backgrounds including ST35 (n = 8), ST14 (n = 4), ST15 (n = 2), ST111 (n = 4), ST234 (n = 4), ST134 (n = 2), ST3887 (n = 2), ST116 (n = 1), ST151 (n = 1), ST431 (n = 1), ST1916 (n = 1), ST2861 (n = 1), ST3030 (n = 1). CRISPR-Cas was significantly more common in CS-*Kp* strains compared with CR-*Kp* strains (*p* = 0.0072, two-sided T test). Antibiograms of the 35 CRISPR-Cas positive CS-*Kp* strains (11.5%) (Fig 2C) were compared with antibiograms of a random sample of non-ST258 CS-*Kp* that lacked CRISPR-Cas (n = 47) as well as to all CS-*Kp* of ST258 clonal background (n = 5) (Table 2). As expected, all 35 CRISPR-Cas positive *Kp* strains were sensitive to carbapenems and resistant to ampicillin (resistance is chromosomally encoded). Additionally, CRISPR-Cas positive strains, all non-ST258, exhibited retained sensitivity to most of the other antibiotics (30/33, 91%) when compared with non-ST258, CRISPR-Cas negative strains (31/47, 66%) (*p*-value = 0.015). Finally, it is notable that a majority of ST258 CS-*Kp* strains (4/5, 80%) exhibited resistance against multiple antibiotics, including amikacin, aztreonam, ceftazidime, ceftriaxone, ciprofloxacin, levofloxacin and tobramycin. Indeed, only one of the CS-ST258 strains, SBU#53, was pan-sensitive to antibiotics, with the exception of the chromosomally encoded ampicillin resistance.

**Table 2:** Antibiogram analysis of CS-*Kp* strains. Numbers and percentages represent non-susceptibilities for that specific antibiotic.

	CS, CRISPR-Cas positive (n = 33), n (%)	non-ST258 CS, CRISPR-Cas negative (n = 47), n (%)	ST258 CS, CRISPR-Cas negative (n = 5), n (%)
Amikacin	0 (0)	3 (6.4)	4 (80)
Ampicillin/Sulbactam	3 (9.1)	11 (23.4)	3 (60)
Aztreonam	1 (3.0)	8 (17.0)	4 (80)
Cefepime	0 (0)	7 (14.9)	2 (40)
Ceftazidime	1 (3.0)	7 (14.9)	4 (80)
Ceftriaxone	0 (0)	9 (19.1)	4 (80)
Ciprofloxacin	0 (0)	4 (8.5)	4 (80)
Gentamicin	0 (0)	4 (8.5)	1 (20)
Levofloxacin	0 (0)	4 (8.5)	4 (80)
Piperacillin/Tazobactam	1 (3.0)	4 (8.5)	1 (20)
Tigecycline	0 (0)	1 (2.1)	0 (0)
Tobramycin	0 (0)	5 (10.6)	4 (80)
Trimethoprim/sulfamethoxazole	2 (6.1)	9 (19.1)	3 (60)

### CRISPR-Cas inhibits transformation of *bla*^KPC^ plasmids in CS-*Kp*

**Fig 3:**
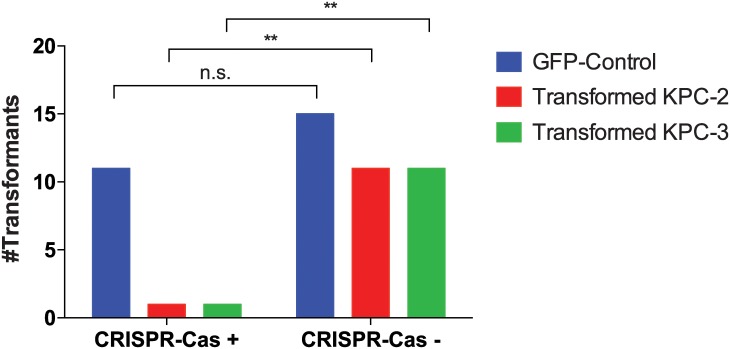
Plasmid transformation assays. Transformation of *bla*_KPC-2_ and *bla*_KPC-3_ plasmids was significantly less successful in CRISPR-Cas positive strains compared with CRISPR-Cas negative strains.

Next, transformation studies were performed to explore whether the presence of CRISPR-Cas protects against the acquisition of *bla*_KPC_ plasmids in CS-*Kp* strains. These experiments showed successful transformation with a control plasmid in 11 of 20 CS-*Kp* strains with CRISPR-Cas (Fig 3). However, of these 11 strains, only 2 (18%) could be transformed with either the *bla*_KPC-2_ (SBU#63) or the *bla*_KPC-3_ (SBU#20) plasmid (but not both) (Fig 3). In contrast, 15 of 20 CS-*Kp* isolates that lacked CRISPR-Cas sequences were successfully transformed with the control plasmid and 11 of those (73%) were successfully transformed with both *bla*_KPC-2_ and *bla*_KPC-3_. Hence, these data indicate higher transformation success of *bla*_KPC_ plasmids in strains lacking CRISPR-Cas compared with strains containing CRISPR-Cas (Fisher’s exact test, *p*-value = 0.0017 for both plasmids). No significant difference in control plasmid transformation was observed between groups. We also attempted to transform all CS ST258 strains (n = 5) with a control plasmid and *bla*_KPC_. Since kanamycin resistance was required for the selection of transformed bacteria with this control plasmid, only 2 of 5 isolates could be studied in this experiment (SBU#53 and SBU#181) as the rest were kanamycin resistant. Both strains were able to be transformed with *bla*_KPC-2_, but not with *bla*_KPC-3_.

### CRISPR spacer sequences in strains protected from plasmid acquisition match other multidrug-resistant plasmids

The CRISPR spacer sequences in strains that could not be transformed with *bla*_KPC-2_ and *bla*_KPC-3_ plasmids were further analyzed. Specifically, we explored whether spacer sequences in the CRISPR array of these strains contained genomic sequences homologous to known multi-drug resistant plasmids. CRISPR in these strains was amplified and then sequenced via a PCR primer set generated from a previously published CRISPR-Cas database of 55 *Kp* strains (18). These PCR primers amplify all described *Kp*-CRISPR-Cas architectures [24] (Fig 1).

PCR was performed on 7 of the CRISPR-Cas positive strains (SBU #28, 31, 61, 63, 70, 83, and 142), all of which were resistant to transformation with *bla*_KPC_ plasmids. In each of these strains, sequence data identified the CRISPR-Cas system as I-E* CRISPR type [24] (Fig 1). Strains SBU #28, 70, and 142 had I-E* CRISPR2 and I-E* CRISPR3, whereas strains SBU #31, 61, 63 and 83 contained only I-E* CRISPR2. The I-E* CRISPR2 and I-E* CRISPR3 direct repeat sequences were found to be identical to the previously reported consensus sequences [24]. Each CRISPR-Cas positive strain contained between 3 and 11 spacer sequences. Spacer sequences exhibited an 87–100% match to exogenous DNA from either known plasmids or phages [28]. Notably, at least one spacer sequence in each of the clinical isolates analyzed either partially or fully matched plasmids (89–100% identity) that were reported to confer resistance to one of the following: metals, non-carbapenem antibiotics, carbapenem antibiotics via *bla*_KPC_ genes plasmids, or carbapenem antibiotics via *bla*_NDM_ genes (Table 3).

**Table 3:** A list of CRISPR sequence identities in each strain analyzed.

Strain	CRISPR spacer #	Identity %	Plasmid	Resistance
SBU#28	2	94	pKPN-498	Heavy metals
SBU#31	1	100	pNJST258N1	Heavy metals
SBU#61	2	94	pKPN-498	Heavy metals
SBU#63	2	89	pKPC-224e	*bla*_KPC-3_
SBU#63	2	89	pKQPS142a	Heavy metals
SBU#70	5	94	pKPN-498	Heavy metals
SBU#70	6	91	pKN-LS6	Heavy metals
SBU#83	1	96	pKPC-c9fd, pKPC-4b66	*bla*_KPC-2_
SBU#142	2	100	pKPC-224e	*bla*_KPC-3_
SBU#142	3	100	pCREC-TJ2-NDM	*bla*_NDM_
SBU#142	6	100	pKPC2_095132	*bla*_KPC-_^2^

### With deletion of CRISPR spacer sequences, transformation was enabled in *Kp* strain #61

Attempts were made to delete the I-E* CRISPR2 spacer sequences in the 7 above described CRISPR-Cas positive strains (using homologous recombination, as this CRISPR2 was present in both I-E and I-E* strains). Inherent resistance of some strains to the pRedET plasmid permitted successful deletion of the I-E* CRISPR2 spacer in only strains SBU#28, SBU#61, and SBU#70. Once the I-E* CRISPR2 spacer was deleted, only SBU#61 was successfully transformed with *bla*_KPC-2_ and *bla*_KPC-3_ plasmids. Notably, SBU#61 only carries I-E* CRISPR2, whereas SBU#28 and SBU#70 also carry I-E* CRISPR3.

## Discussion

This study determined the prevalence of CS-*Kp* in ST258 colonized patients in one major hospital in New York State. CR-*Kp* was responsible for 15.5% of *Kp* infections in our patient population, which is slightly below that reported in three NYC hospitals between 2006–2014 (17.3%) [29]. ST258 was responsible for 85.1% of the CR-*Kp* in our patient population, which is higher than previously reported [4, 7]. Additionally, this study determined the prevalence of CRISPR-Cas in CR-*Kp* and CS-*Kp* for both ST258 and all other strains tested. CRISPR-Cas was only identified in CS-*Kp* strains that were not of the ST258 clonal background, which has been reported by others [30–32]. Overall the proportion of strains with CRISPR-Cas systems in our isolate population was only 11.5%, consistent with data of other analyzed *Kp* genomes, but much lower than data derived from *Escherichia coli* isolates [33]. This difference could be related to the species itself or to technical differences in our studies, as our approach detected only the presence of Cas proteins (full CRISPR-Cas systems). As the CRISPR-Cas system appears not to be highly prevalent in *Kp* species, it remains to be determined how the absence or presence of the system could be contributing to the evolution of *Kp* strains.

Our data describe CS strains that belong to the ST258 clonal background that lacked CRISPR-Cas systems an. Of these strains 80% strains exhibit other drug resistance. Future in depth characterization of the sole pan-sensitive CS ST258 strain identified in this study, and others if identified, might help explain why some strains do not acquire antibiotic resistance. Ultimately, it would be interesting to know what, if any, fitness advantage there is for pan-sensitive strains.

Most of the *Kp* strains containing CRISPR-Cas were pan-sensitive whereas resistance to multiple antibiotics was observed in the strains lacking CRISPR. In line with this, our transformation studies demonstrated successful transformation of the *bla*_KPC-2_ and *bla*_KPC-3_ plasmids into a significantly larger proportion of *Kp* strains lacking CRISPR-Cas. Although some strains could not be transformed with the control plasmid, successful control plasmid transformation was comparable among strains with and without CRISPR-Cas systems. The impedance of transformation may have been due to transformation competency preparation restriction-modification systems inherent to the strains or incompatibility (Inc) groups if the strains harbored other plasmids.

Notably, transformation of the *bla*_KPC-2_ plasmid was successful in only 1 CS-*Kp* strain containing CRISPR-Cas (SBU#63), as was transformation of the *bla*_KPC-3_ plasmid (SBU#20). Interestingly, the former strain had a CRISPR-Cas spacer sequence that matched a published *bla*_KPC-3_ plasmid. The ability to be transformed with one plasmid but not the other could be related to the CRISPR-associated spacer sequences that match either *bla*_KPC-2_ or *bla*_KPC-3_ plasmids, but not both, as is the case in SBU#63. Sequence analysis of spacers in 7 CRISPR-Cas positive strains indicated that they contained sequences that were more than 90% identical to sequences found in multidrug-resistance plasmids carrying either carbapenemase genes or resistance genes against heavy metals. CRISPR-Cas and its associated spacer sequences could therefore help explain why some *Kp* strains are less susceptible to the acquisition of antibiotic-resistance plasmids.

Furthermore, after successful deletion of the I-E* CRISPR2 region in strain SBU#61, which previously could not be transformed with any bla_KPC_ plasmids, transformation of this strain with *bla*_KPC-2_ and *bla*_KPC-3_ plasmid was successful. This supports the notion that CRISPR sequences initially shielded this strain from acquiring carbapenem resistance by preventing integration of the *bla*_KPC-2_ and/or *bla*_KPC-3_ plasmid. At this point it remains unclear why transformation with *bla*_KPC-2_ and or *bla*_KPC-3_ was not successful in other tested strains SBU#28 or SBU#70, even though the CRISPR cassette was also deleted. It is important to note, however, these strains contained CRISPR3 spacers, and our deletion strategy only targeted the CRISPR2 locus. This CRISPR3 sequences may still target sequences in the plasmids, preventing the transformation.

One major limitation of our data is that we do not have the complete sequences of the *bla*_KPC-2_ and *bla*_KPC-3_ plasmids of our *Kp* strains, and, in addition, we did not do transformation studies using other plasmids identified to have high identity to the spacer regions. As a result, we cannot conclusively demonstrate that CRISPR-Cas spacers convey immunity due to similarity of the CRISPR spacers to plasmid sequences. Lack of transformation success could also be due to the presence of other plasmids that share the same incompatibility system with the *bla*_KPC_ plasmids, although 6 of the 7-sequenced CRISPR-Cas positive were pan-sensitive to all antibiotics pointing against that line of thought. Finally, these results, while based on limited data from 7 CRISPR-Cas positive strains, do differ from a previous analysis of CRISPR-Cas in *E*. *coli* isolates where the proportion of spacers matching plasmid genomes was found to be low (20%), and there was no association between CRISPR-Cas and antibiotic resistance [34].

PCRs done to amplify CRISPRs sequences assigned all of our strains to the I-E* CRISPR type, a subtype of the *Kp* I-E CRISPR type [24]. It would be interesting to investigate whether phenotypic or geographic differences exist between strains harboring different CRISPR types.

A limitation of this study is that this study only analyzed clinical isolates from SBUH and results may therefore not reflect the transmission or characteristics of *Kp* strains and plasmids present in other geographical areas. Future studies are needed to investigate why some ST258 strains remain sensitive to carbapenems and whether these strains compensate by having other fitness advantages or virulence genes. It is possible that the ST258 clone evolved rapidly in the past in a selective pressure environment (i.e. the clinical setting) without the CRISPR-Cas system as a barrier against acquisition of exogenous DNA. Although whole genome sequence studies suggested that ST258 spread via local expansion rather than by repeated uptake of multiple antibiotic-resistance plasmids, acquisition of novel virulence genes has been described in ST258. This acquisition remains a concern for other *Kp* clones as well because it may contribute to the evolution of hypervirulent, multidrug-resistant strains [35, 36]. While further studies need to be done, decreased prevalence of the CRISPR-Cas system in the ST258 clonal background may be a major contributor to its clonal success, acquisition of antibiotic resistance plasmids and virulence genes, hospital outbreaks, and global spread.

## Supporting information

S1 Fig Flow diagram of the methods used for identification of ST258 strains. (EPS)

S1 Table Minimal data set. (XLSX)
